# Risk Assessment to Evaluate if Crayons Complying with the Consumer Product Safety Improvement Act of 2008 for Lead, Also Comply with California Proposition 65

**DOI:** 10.3389/fpubh.2017.00130

**Published:** 2017-06-02

**Authors:** Gulzar R. Ahmad, Subodh Kumar, Dildar Ahmad, Masood A. Shammas

**Affiliations:** ^1^InfoTox International Inc., Riverside, CA, United States; ^2^Department of Adult Oncology, Harvard (Dana Farber) Cancer Institute, Boston, MA, United States

**Keywords:** crayons, lead, California proposition 65, Consumer Product Safety Improvement Act, compliance, cancer, reproductive disorders

## Introduction

The purpose of the legislation of the California Proposition 65, officially known as the Safe Drinking Water and Toxic Enforcement Act of 1986, was to protect the citizens and the drinking water of California state and to inform citizens about chemicals, which are associated with reproductive disorders, birth defects, and cancer. The part of statute states that “no person in the course of doing business shall knowingly and intentionally expose any individual to a chemical known to the state (California) to cause cancer or reproductive toxicity without first giving a clear and reasonable warning…” (1).

Crayons are among widely used substances, especially by children. Even if we take our children to a restaurant in western countries such as USA, the first thing they get on the table is a set of crayons. Although in general they are not toxic and are less messy than some of other art materials, they can have varying levels of lead contamination (2–4). Lead is a toxic heavy metal and its exposure poses a major health risk to consumers (5–7). Lead is non-biodegradable, which is a major cause of its prolonged perseverance in the environment. Exposure to lead causes numerous harmful effects on various organs including nervous and reproductive systems (7). Lead exposure has been associated with impaired spermatogenesis, reduced serum testosterone level, infertility, and abnormal prostatic functions in males (7, 8) and infertility, miscarriage, and pregnancy hypertension and premature delivery in females (9, 10). Among all the organs, the nervous system is the key target for lead-induced toxicity. Lead is associated with many neurological disorders including nerve and brain damage, Low IQ, delayed growth, short-term memory and hearing loss, behavioral problems, and possibly diseases such as Parkinson’s disease, Alzheimer’s disease, and schizophrenia (7, 11). Lead poisoning in children, even at low level, significantly affects their nervous system. This can cause reduced IQ, lack of ability to concentrate, slowed growth, irritability, and hyperactivity of the child. Although, not specifically related to children, lead exposure has also been associated with a variety of genomic changes including deletions, chromosomal gaps, fusion, and polyploidy (12, 13). There are also reports that lead induces aberrant DNA repair, which causes chromosomal aberration (14). Some studies also suggest that chronic exposure to lead may cause increased DNA damage and aberrant DNA repair leading to carcinogenesis (6, 7). Data from our laboratory also demonstrate that dysregulated DNA repair is a prominent mechanism underlying ongoing genomic evolution of cancer cells (15, 16) and development of resistance to treatment (15). Crayons could have varying levels of lead (2). More importantly, Rastogi and Pritzl (3) have reported the migration of a considerable amount (ranging from 0.03 to 24.27 ppm) of lead from crayons. Therefore, exposure to this or even a lower amount of lead over a long period of time, especially in combination with other extrinsic and intrinsic factors [including other heavy metals/chemicals, radiation, and lifestyle factors (17)] could potentially pose a great health risk. This is because toxicity of a heavy metal could become much higher when combined with other metals (18, 19) or agents with similar biochemical properties.

There has been a constant effort to reduce the exposure to lead (5). Although much progress has been made, there is need to further minimize the exposure to ensure safety for consumers (20). Since crayons are among the most widely used products by children and can potentially be contaminated with lead, their formulations have to comply with both the Consumer Product Safety Improvement Act (CPSIA) of 2008 as well as California Proposition 65. According to Consumer Product Safety Commission (CPSC) guidelines, the amount of lead in accessible parts of the products used by children cannot be more than 100 ppm (21). The purpose of this article was to evaluate if crayons complying with the CPSIA of 2008 for lead, also comply with California Proposition 65.

## Methods and Results

We based our assessment on assumption that a child ingests 14 g of crayon material per month from a package of 12 crayons. Using this assumption, we assessed the risk of exposure to a child in terms of average exposure per day over entire lifetime and compared this with the guidelines set by CPSIA and Prop 65. We made another assumption that a child will play with crayons for a period of 3 years (from 3 to 6 years of age) during his/her expected life expectancy of 70 years. Since products used by children cannot have more than 100 ppm lead, we used 99 ppm (i.e., <100 ppm) as acceptable level for our calculations.

1 ppm = 1 mg of something per kilograms (mg/kg or mg/1,000 g). Therefore, we can calculate that at the concentration of 99 ppm of lead in crayons, how much lead a child could ingest from 14 g of crayons over the period of 30 days:

$$\begin{array}{ll} \left(99/1,000\right)*14 & =1.386\text{ mg or}\\ \; & =1,386\text{ μg} \end{array}$$

Over the period of 3 years (or 36 months), this amount will become:

1,386×36=49,896 μg

When equalized over the life expectancy of 70 years, the daily exposure among children playing with the crayons is calculated to be:

49,896/70=712.8 μg/year

From this, we can calculate exposure per day:

712.8/365=1.95 μg/day (approximately).

This level is ~8-fold less than a no-significant-risk-level for carcinogens (15 μg/day; oral) and ~4-fold more than a maximum allowable dose level for reproductive toxins (0.5 μg/day), established by the State of California.

## Conclusion and Recommendations

Based on the assumptions described above, we conclude that the crayons containing 99 ppm of lead, a level at which the crayons comply with the CPSC regulation under the CPSIA, will require labeling under the California Proposition 65, the Safe Drinking Water and Toxic Enforcement Act of 1986, as a reproductive toxin. Lead is a toxic heavy metal and poses serious health risks including abnormalities of male and female reproductive systems (4–7), neurological disorders (4, 8), DNA damage, and chromosomal abnormalities (9, 10) leading to carcinogenesis (3, 4). To minimize the risk to children, the CPSC may also consider further lowering the permissible level of lead in products for children. To produce safer products for children, manufacturers should also lower the level of lead in crayons. Moreover, to minimize the risk of law suits under California Prop 65, manufacturers and distributors should also test their crayons for lead to assure compliance to California Prop 65. California’s Prop 65 has a “citizen lawsuit” provision that encourages private citizens to file lawsuits against businesses they claim are not fully complying with the law—regardless of whether or not that is true.

## Author Contributions

GA identified the problem and contributed in preparation of manuscript; SK assisted in scientific explanation of the problem, highlighted mechanisms involved in associated health risks, and contributed in manuscript preparation; DA provided critical toxicological expertise and assisted in critical evaluation of problem and manuscript preparation; MS supervised the process, integrated and analyzed information, and prepared the manuscript.

## Conflict of Interest Statement

The authors declare that the research was conducted in the absence of any commercial or financial relationships that could be construed as a potential conflict of interest.
